# Comparative Study on the Effects of Ginseng and Green Tea Extracts on Selected Lipid Metabolism Markers in Adipocyte

**DOI:** 10.5812/ijpr-162445

**Published:** 2026-02-09

**Authors:** Mandana Salehi, Kahin Shahanipour, Ramesh Monajemi, Parisa Mohamadynejad

**Affiliations:** 1 Department of Biochemistry, Fal.C., Islamic Azad University, Isfahan, Iran; 2 Department of Biology, Fal.C., Islamic Azad University, Isfahan, Iran; 3 Department of Biology, ShK.C., Islamic Azad University, Shahr-eKord, Iran

**Keywords:** Ginseng, Green Tea, Lipid Metabolism, BMP7, HSL, Leptin

## Abstract

**Background:**

Dietary patterns are effective in obesity treatment. This has led to more investigations on its mechanisms in combating obesity.

**Objectives:**

This study investigated the effects of ginseng and green tea extracts (GTE) on selected markers of lipid metabolism and the expression of some related genes in adipocytes.

**Methods:**

After a one-month period of consuming a high fatty content diet, a total of 42 male Wistar rats were assigned to seven groups randomly. The rats were then subjected to an eight-week treatment where they were administered different dosages of GTE and ginseng extract (GE) through oral administration. Then, some serologic parameters pertaining to lipid metabolism were analyzed in the treated rats. Furthermore, alterations in the expression levels of select genes, bone morphogenetic protein 7 (BMP7), hormone-sensitive lipase (HSL), and leptin, implicated in lipid metabolism, were quantified within the adipose tissue of the rats utilizing the reverse transcription-quantitative polymerase chain reaction (RT-qPCR) methodology. Ultimately, the chemical composition of the extracts was analyzed by high performance liquid chromatography (HPLC).

**Results:**

The findings indicated that the utilization of the extracts had a notable impact on the reduction of body weight. There was a noteworthy enhancement of high-density lipoprotein levels across all study groups, as indicated by a statistically significant increase at a confidence level of 95%. The efficacy of the administered extracts was observed in a significant upregulation in BMP7 and HSL gene expression. Conversely, there was a notable reduction in leptin expression, which reached statistical significance at a confidence level of 95%. HPLC results detected 9 ginsenosides in the GE, among which Rb1 (100 mAU) was present in the largest amount, and 9 alkaloids in the GTE, among which epicatechin (EC) (380 mAU) and caffeine (320 mAU) were present in the largest amount.

**Conclusions:**

The present study holds the potential to offer novel insights regarding the mechanism through which GE and GTE exert their anti-obesity effects.

## 1. Background

Obesity is a prevalent community health issue that can arise as a consequence of escalated calorie intake (1). The primary risk factors commonly related to obesity encompass pathological conditions including diabetes type II, hypertension, and atherosclerosis (2). In recent years, there has been a notable surge in the prevalence of obesity among both men and women, with rates increasing at a compounded rate of two to three times over the span of the previous decade (3). Obesity, as an intricate health condition, has been linked to a number of contributing factors such as genetics, lifestyle, and diet (2, 4). Alongside the implementation of chemotherapy, various medicinal herbs are globally employed in the management and mitigation of obesity. For instance, the utilization of ginseng and green tea has been advocated for promoting weight loss. This recommendation stems from their capacity to amplify energy intake and enhance the process of fat oxidation within cellular structures. Specifically, their positive effects are attributed to the reinforcement of gene expression associated with the oxidation of fatty acids, consequently resulting in an elevation of lipolysis (5).

Ginseng exhibits a diverse range of biological activities owing to the presence of its primary bioactive compounds, ginsenosides Rb1 and Rb2. Extensive research has discerned the impact of ginsenosides on obesity while corroborating their physiological attributes, including impediment of lipid accretion and diabetes, as well as enhancement of immune system functionality. Ginsenoside Rb1 induced a reduction in fat content among rats that were nourished with a high-fat diet (HFD), subsequently resulting in weight loss (6). Numerous investigations have been conducted to examine the impact of green tea extract (GTE) on body weight reduction, which have yielded findings indicating that the botanical constituent operates through the inhibition of lipid production, augmentation of lipolysis, induction of apoptosis, prevention of energy absorption, upregulation of genes associated with fatty acid oxidation, and elevation of energy expenditure (7). Flavonoids are one of the most powerful bioactive compounds in ginseng and green tea. The main flavanols which have been reported in ginseng are catechin (46.03 mg/100 g of dry matter), kaempferol (49.61 mg/100 g of dry matter), and quercitrin (9.67 mg/100 g of dry matter) (8). Green tea has been reported to comprise catechins (280 - 580 g/kg dry extract) and caffeine (75 g/kg dry extract) (9, 10).

Various genes play a pivotal role in regulating distinct biological pathways associated with the browning of white adipose tissue (WAT). The process of browning ensues through intricate mechanisms that are attributed to the bone morphogenetic pathway, specifically involving the activation of bone morphogenetic protein 7 (BMP7) and fibroblast growth factor-21 (FGF-21) (11). The EWS/YBX-1/BMP7 (Ewing sarcoma gene/Y box binding protein1/bone morphogenetic protein 7) has been posited as a potential suppressor of the browning process (12). One of the primary transcriptional regulators involved in lipid differentiation is the peroxisome proliferator-activated receptor gamma (PPAR-γ). This receptor activates specific browning-associated genes in a coordinated manner with the PR domain-16 (PRDM-16) containing PPAR-γ coactivator-1α (PGC-1α)-16 (13). The augmentation of the process of browning positively impacts the quantity of WAT by promoting the stabilization of the PGC-1 mediated by FGF-21 (14). In comparison, the transducin-like enhancer protein 3 (TLE-3) is implicated in the negation of regulatory pathways through its ability to stimulate the expression of white-selective genes while concurrently repressing brown-selective genes (15). The disparity between brown adipose tissue (BAT) and WAT is evident through discrepancies in their regulatory genes, as well as in the genes responsible for the oxidation of fatty acids within the mitochondria. The expression levels of carnitine palmitoyltransferase-1 (CPT-1) and cell death activator (CIDEA) are found to be comparatively higher in BAT in contrast to WAT. The regulation of energy intake through appetite is primarily controlled by the hormone leptin, which exerts its effects by binding to the receptors located in the arcuate nuclei of the hypothalamus (16). A research investigation has demonstrated the efficacy of ginsenoside Rb1 in lowering interleukin-6 levels and mitigating the negative regulatory impact on leptin signaling. The consumption of ginseng has been found to yield a reduction in plasma levels of leptin and neuropeptide Y, as indicated by prior research (6). The contemporary way of life contributes to the generation of oxidative compounds that encompass free radicals within the human organism. These products have the potential to contribute to the onset of different diseases, including cancer and diabetes (17, 18).

## 2. Objectives

While prior research has well demonstrated the beneficial effects of ginseng extract (GE) and GTE on obesity, in this approach, for the first time, the simultaneous effects of GE and GTE were investigated on serologic parameters pertaining to lipid metabolism, including those associated with adipocytes BMP7/hormone-sensitive lipase (HSL)-driven browning. We also offer novel insights in the context of white-to-brown adipose transition by BMP7/HSL activation utilizing GE and GTE in rats fed an HFD.

## 3. Methods

### 3.1. Animal Grouping and Fattening Rats

In this investigation, a cohort comprising forty-two male Wistar rats exhibiting a lifespan of 8 weeks and an average weight of 150 ± 2.1 g was subject to random allocation, resulting in the formation of seven distinct groups, each composed of six individuals (N = 6). In order to acclimate animals to controlled laboratory environments, they were subjected to a temperature range of 22 - 25°C and a relative humidity of 45 - 55 within a 12-hour cycle of light and darkness. This condition was maintained for a duration of one week, during which the animals were provided with a regular diet. The control group was provided with a standard diet that consisted of the following nutrient proportions: 20.3% protein, 63.9% carbohydrates, and 15.8% fat. On the other hand, the remaining six groups were subjected to an HFD for a duration of four weeks, characterized by nutrient ratios of 17% protein, 43% carbohydrates, and 40% fat (19). In the subsequent phase, samples of blood were collected from the ocular corners of rats and subsequently, the serum was separated. The serum hyperlipidemia was confirmed in the rats by performing tests including total cholesterol (TC), triglyceride (TG), and low-density lipoprotein (LDL) measurements. During the experiment, every effort was made to minimize any discomfort or distress experienced by the animals, and a humane approach was ensured in all aspects of their care and handling.

### 3.2. Treatment and Sampling

Ginseng and green tea were obtained from Isfahan Plant Import Company, a reputable importer located in Iran. The hydro-alcoholic extracts of ginseng and green tea were prepared via immersion in a solvent consisting of 70% ethanol. The extracts underwent filtration using a 0.22 µm syringe filter (JetBiofil, China). The filtrates were used for treating the rats through oral administration. However, the control group was administered pure normal saline. The control group was provided with a standard diet in accordance with the guidelines described in the preceding section. The remaining groups were administered the following treatments: Group A was administered a daily dose of 77.2 mg/kg of GTE, group B received a dosage of 154.6 mg/kg/day of GTE. Group C received GE with a dosage of 102.8 mg/kg/day and group D received GE with a dosage of 205.6 mg/kg/day. Group E received GTE with a dosage of 154.6 mg/kg/day and GE with a dosage of 205.6 mg/kg/day, simultaneously. This grouping was done based on the 3Rs (replacement, reduction, and refinement) defined by Russell and Burch (20). The conversion of the rat's ingestion dose from the human equivalent dose was conducted using a formula as stipulated by the US Food and Drug Administration (21):


$$Animal\;does\;(mg\;/\;kg)=\frac{Human\;equivalent\;dose\;(mg\;/\;kg)\;}{Animal\;Km\;/\;Human\;Km\;}$$

The correction factor (Km) was estimated by dividing the average body weight (kg) of species by its body surface area (m²). Therefore, the Km factors were calculated as: Human Km factor = 60 kg/1.62 m² = 37, and rat Km factor = 0.15 kg/0.025 m² = 6 (22).

Receiving GTE at a concentration of 12.5 mg/kg/day has been reported to significantly reduce body weight (23). According to the above, about 77.2 mg/kg per day of GTE for a rat is equivalent to 750 mg/day for a 60 kg human. Therefore, in the present study, 77.2 mg/kg/day and 154.6 mg/kg/day of GTE were used. Administration of 102.8 mg/kg per day of GE for a rat is equivalent to 1000 mg/day for a 60 kg human (6, 24). Therefore, in the current study, 102.8 mg/kg/day and 205.6 mg/kg GE were used.

Throughout the experimental protocol, the subjects were administered a conventional diet. After a period of eight weeks, a reevaluation was carried out to assess the weight and Body Mass Index (BMI) of the rats. The rats were restrained manually for the minimum duration necessary and then administered an anesthetic solution, comprising ketamine-xylazine (KX), at a dosage of 0.7 ml per 100 g of body weight. The parameters including depth of anesthesia, body temperature, respiratory status (depth, rate, and pattern), and heart rate were monitored during anesthesia. Afterward, blood samples were collected from the cardiac region of the rats, and the adipose tissue of the rats was isolated and stored at a temperature of -70°C. The animal experimentation was carried out in accordance with the protocol approved by the Animal Welfare and Use Committee. Random number generation was used for treatment randomization and investigators involved in performing treatments and data collection did not know which participants received which treatments and the outcome of measurements.

### 3.3. Lipid Profile and Biochemical Analysis

The collected blood serum was subjected to centrifugation at a speed of 4000 g for a duration of 15 min at a temperature of 4°C. Total cholesterol (TC), triglyceride (TG), high-density lipoprotein (HDL), low-density lipoprotein (LDL), and very low-density lipoprotein (VLDL) were measured using special diagnostic kits (Pars Azmoun Co. Iran) based on the enzyme-calorimetric method by Hitachi 1000 autoanalyzer.

### 3.4. Realtime Reverse Transcription-Polymerase Chain Reaction Analysis for Bone Morphogenetic Protein 7, Hormone-Sensitive Lipase, and Leptin Genes Expression Analysis

Total RNA extraction from the adipose tissue was carried out utilizing RNXplus buffer (Sinaclon, Iran) as instructed in the manufacturer manual. The initial complementary DNA strand was produced utilizing the AddBio cDNA synthesis kit (South Korea), adhering to the guidelines provided by the manufacturer. Relative quantitative reverse transcription-polymerase chain reaction (RT-qPCR) was conducted to assess alterations in the expression levels of BMP7, HSL, and leptin genes (21). Specific primers for target genes were constructed using Generunner V6.554X64 beta software. The comprehensive catalogue including the primers and their respective properties is presented in Table 1. The ActB gene is commonly utilized as an internal control gene in molecular studies. To optimize the reaction conditions, a final concentration of 1X of SYBR green 2X master mix (manufactured by Takara Co., Iran) was utilized. In this study conducted in Japan, a concentration of 0.4 µM was employed for both forward and reverse primers, while approximately 50 ng of cDNA was utilized as the template. The thermal program consisted of an initial denaturation step at 95°C for a duration of 10 min, followed by 40 cycles comprising a denaturation step at 95°C for 15 seconds, an annealing step at 55°C for 30 seconds, and an extension step at 72°C for 30 seconds. The melting curve was determined within the temperature range of 65 - 95°C.

**Table 1.** The Primers that Used for the Amplification of Target Genes in the Study

Accession Number in GenBank and Primers	Sequences	Amplicon Length (bp)	Target Genes
**NM-031144.3**		109	ActB
ACTB-F	5’- AGCCTTCCTTCCTGGGTATGG-3’		
ACTB-R	5’ AGCACTGTGTTGGCATAGAGG-3’		
**NM-012859.1**		131	HSL
HSL-F	5’- GAAGGGCAGGACAGCAAGATG-3’		
HSL-R	5’- ACAAAGCCACCACCGTGAATG-3’		
**NM-001191856.2**		138	BMP7
BP7-F	5’- ACACAGGCAGGGAGTCCGAC-3’		
BP7-R	5’ - GGTGTAAGCCCAGGTTGTGC-3’		
**NM-013076.3**		133	Leptin
LEP-F	5’-CACACACGCAGTCGGTATCC-3’		
LEP-R	5’-GGCAAGCTGGTGAGGATCTG-3’		

Abbreviations: BMP7, bone morphogenetic protein 7; HSL, hormone-sensitive lipase.

### 3.5. High Performance Liquid Chromatography

The chemical compositions in hydro-alcoholic extracts of ginseng and green tea were analyzed by HPLC (Agilent 1100 series; Agilent Technologies, Santa Clara, CA, USA) on an Agilent ZORBAX SB-Aq C18 column (4.6 mm × 250 mm, 5 μm). A ginsenoside standard mixture consisting of Rb1, Rb2, Rc, Rd, Re, Rf, Rg1, Rg3, and CK (Advanced Chemical, Taiwan) was used. The utilized solvents were acetonitrile (A) and ultra-pure water (B). A total of 10 μL of the sample was injected into the apparatus, and the column temperature was adjusted to 30°C. The flow rate was set at 1.0 mL/min and the detection wavelengths were set at 203 nm (25) and 270 nm (26) for ginseng and GTEs, respectively. A program included 30% solvent A and 70% solvent B (t = 0 min) to 100% solvent A at t = 70 min (25, 26). The results were expressed as milli absorbance unit (mAU).

### 3.6. Data Analysis

The data were subjected to analysis through the employment of the one-way ANOVA method with the utilization of SPSS v.22. Since a small sample size was provided in this study, a Shapiro-Wilk test was used and showed that the distribution of X departed significantly from normality (W = 0.94, P-value < 0.01). Levene’s test also was used in order to test the homogeneity of variances. The results showed that the P-values were greater than 0.05 and there were no significant differences between the variances. The results were presented as the average error. The statistical significance at confidence levels of 95% and 99% were considered in this study. The Bonferroni correction was used in statistics to adjust the significance level. Additionally, the GraphPad Prism 9.0 software was utilized for the creation of graphical designs.

## 4. Results

### 4.1. Body Mass Index and Food Intake

**Figure 1.**
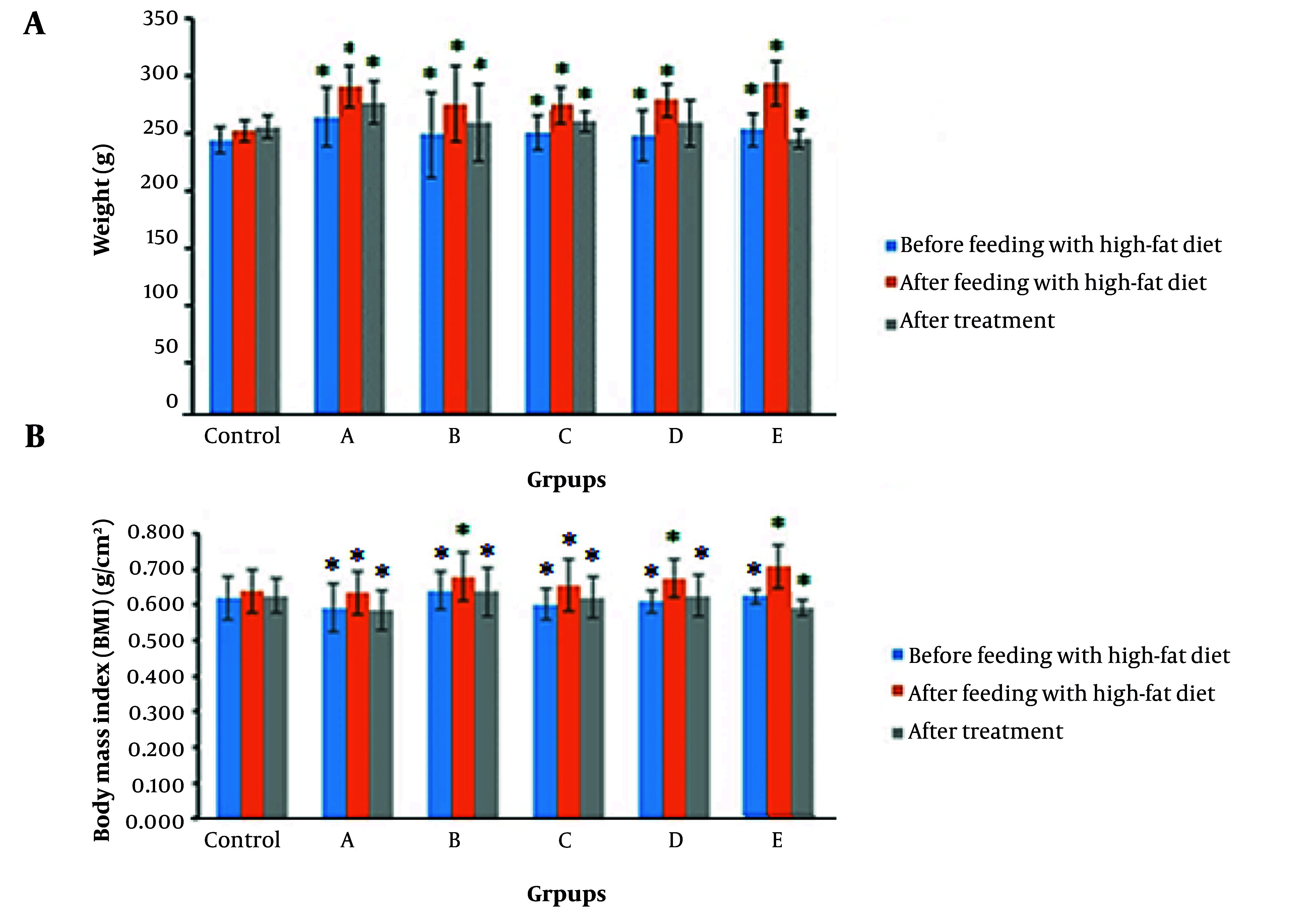
A, evaluating the body weight (g) of rats at three times: before feeding with high-density lipoprotein (HDL), after feeding with HFD, and after treatment. Results showed that after feeding with an HFD, the weight of rats increased in all groups compared to the control group (P < 0.05), and after treatment with the extracts, decreased weight was seen which was significant (P = 0.03 for group A, P = 0.042 for group B, P = 0.03 for group C, P = 0.021 for group D, and P = 0.04 for group E). This decrease occurred more in group E than in other groups (P = 0.02). B, evaluating the Body Mass Index (BMI) (g/cm²) of rats at three times: before feeding with HFD, after feeding with HFD, and after treatment. Results showed that after feeding with an HFD, the BMI of rats increased in all groups compared to the control group (P < 0.05), and after treatment with the extracts, decreased BMI was significant (P = 0.029 for group A, P = 0.032 for group B, P = 0.03 for group C, P = 0.02 for group D, and P = 0.019 for group E). This decrease occurred more in group E than in other groups (P = 0.024). * indicates a significant difference at P < 0.05.

HFD resulted in a significant elevation of the BMI in comparison to the control group. In contrast, the post-treatment evaluation of BMI demonstrated a notable reduction in BMI values compared to the control group, especially in group E (15.7 ± 1.18%). Weight loss was detected across all experimental groups; notably, group E exhibited a greater amount of weight loss (17.2 ± 1.29%) compared to the control group (Figure 1A and B). The extract with utilized dosages did not lead to abnormal behavior and mortality in the experimental groups in comparison to the control group.

### 4.2. Serum Biochemical Parameters

**Figure 2.**
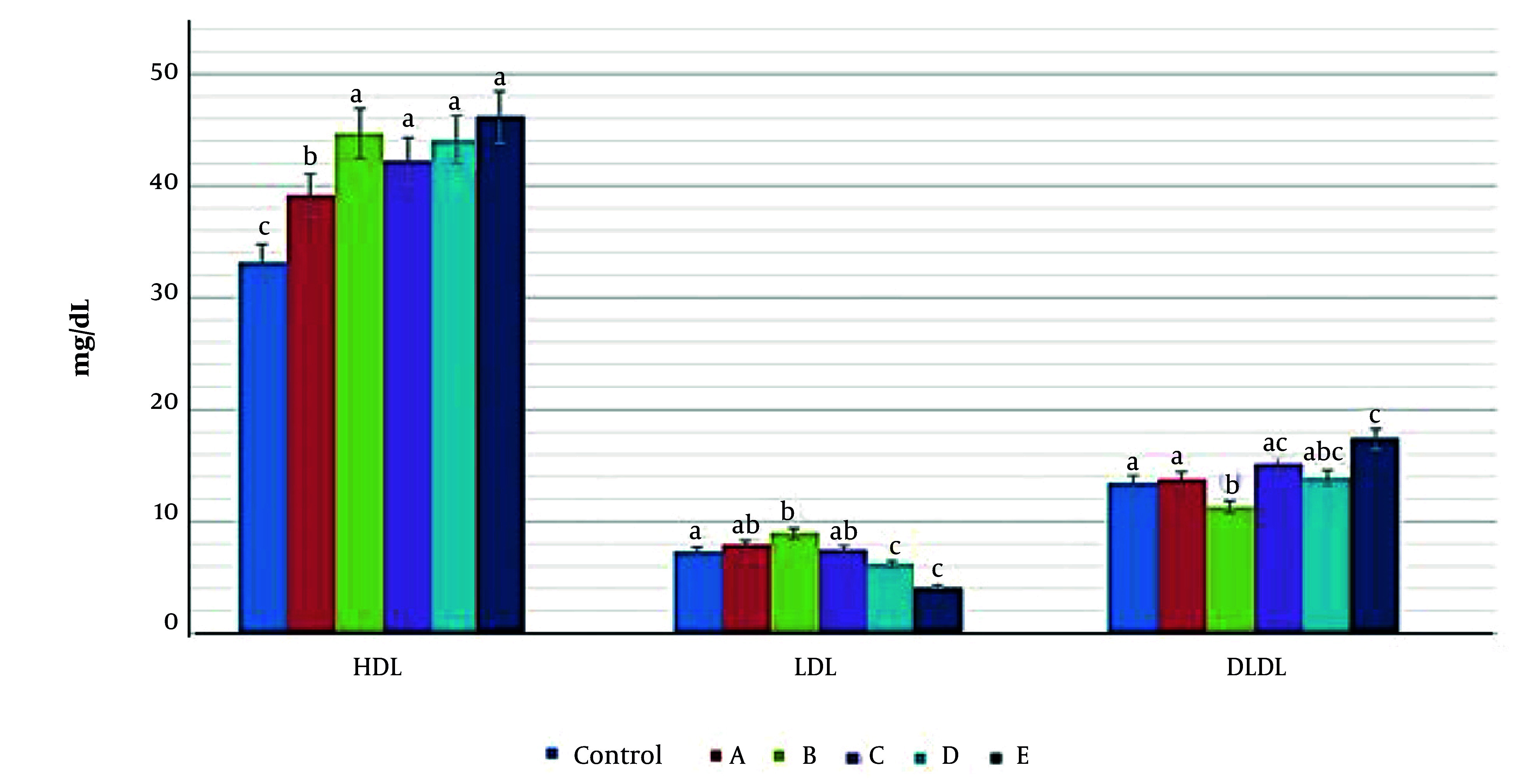
Evaluation of high-density lipoprotein (HDL) (mg/dL), low-density lipoprotein (LDL) (mg/dL), and very low-density lipoprotein (VLDL) (mg/dL) levels in different experimental groups. The highest increase in HDL levels was seen in groups B, C, D, and E compared to the control group (P = 0.031, P = 0.033, P = 0.03, and P = 0.029, respectively) and a slight but significant increase in HDL was seen in group A compared to the control group (P = 0.042). Slight increased level of LDL was seen in groups A and B compared to control (P = 0.045, P = 0.021, and P = 0.333, respectively) and in groups C and E a slight decrease was seen compared to control (P = 0.021, and P = 0.019, respectively). In the case of VLDL, increased levels were observed in groups A, C, D, and E (P = 0.046, P = 0.022, P = 0.032, and P = 0.015, respectively) and a decrease was seen in group B (P = 0.023). Different letters indicate significant differences between treatments.

The serum biochemical parameters, namely triglycerides (TG), low-density lipoprotein (LDL), very low-density lipoprotein (VLDL), total cholesterol (TC), and high-density lipoprotein (HDL), were investigated in order to ascertain the effects of an HFD on rat blood composition. A comparison was made between the values obtained from the experimental group and a control group both before and after treatment. The findings revealed that consumption of an HFD led to an elevation in the levels of TG, LDL, and TC in the blood of rats. However, following treatment, these parameter values decreased to match those of the control group. The augmentation in HDL levels following treatment with extracts (39 ± 1.1 mg/dL, 45 ± 3.2 mg/dL, 42 ± 1.9 mg/dL, 44 ± 2.8 mg/dL, and 46 ± 3.1 mg/dL, in groups A, B, C, D, and E, respectively), as compared to the control group (33 ± 2.1 mg/dL), signifies the beneficial impact of these extracts on the equilibrium between HDL and LDL levels, (8 ± 0.2 mg/dL, 9 ± 1.1 mg/dL, 7 ± 0.5 mg/dL, 6 ± 1.0 mg/dL, and 4 ± 0.1 mg/dL, in groups A, B, C, D, and E, respectively), compared to the control group (7 ± 1.0 mg/dl). This effect was greater in group E than the other groups (Figure 2). 

### 4.3. Expression Level Changes of Bone Morphogenetic Protein 7, Hormone-Sensitive Lipase, and Leptin Genes

**Figure 3.**
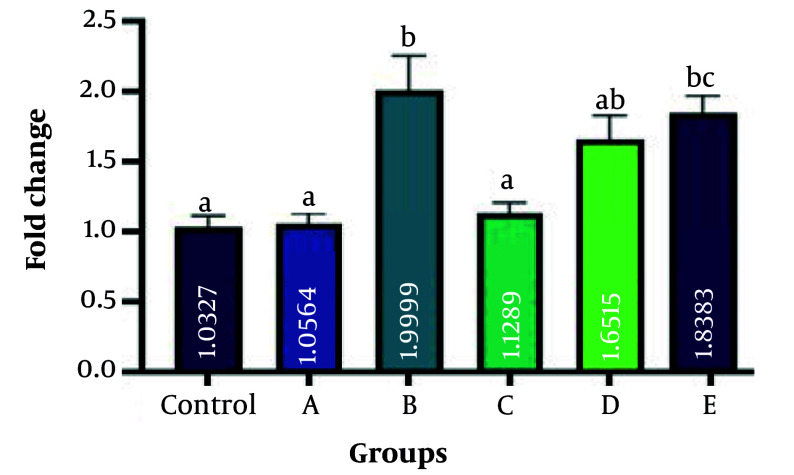
Bone morphogenetic protein 7 (BMP7) gene expression changes. Bone morphogenetic protein 7 gene expression was significantly increased in groups B, D, and E compared with the control group (P = 0.004, P = 0.039, and P = 0.001, respectively). The rate of gene expression changes in groups A and C was insignificant compared to the control group (P = 0.284, and P = 0.301, respectively). Different letters indicate significant differences between treatments.

The present study employed RT-qPCR to measure the expression levels of BMP7, HSL, and leptin genes. These genes are believed to play a role in the conversion process of WAT into BAT. The investigation aimed to assess the impact of ginseng and GTEs on this adipose tissue transformation. Figures 3, , 4, and 5 display the results obtained from this analysis. Bone morphogenetic protein 7 gene expression was significantly increased in groups B, D, and E (fold changes 2 ± 0.02, 1.65 ± 0.01, and 1.8 ± 0.11, respectively) compared with the control group (P = 0.004, P = 0.039, and P = 0.001, respectively). The rate of gene expression changes in groups A and C (fold changes 1.1 ± 0.01 and 1.15 ± 0.01, respectively) was insignificant compared to the control group (P = 0.284, and P = 0.301, respectively). hormone-sensitive lipase gene expression was significantly increased in groups B, C, D, and E (fold changes 1.7 ± 0.21, 1.8 ± 0.11, 1.2 ± 0.11, and 2.1 ± 0.21, respectively) compared with the control group (P = 0.003, P = 0.003, P = 0.022, and P = 0.002, respectively). No difference in gene expression was observed in group A (fold change 0.7 ± 0.02) compared to the control (P = 0.42). Evaluation of leptin gene expression showed the highest expression in groups D and E (fold changes 3.0 ± 0.21 and 2.8 ± 0.11, respectively) compared to the control group (P = 0.005 and P = 0.006, respectively). No difference in gene expression was observed in groups A, B, and C (fold changes 0.9 ± 0.11, 1.15 ± 0.01, and 0.85 ± 0.09, respectively), compared to the control (P = 0.052, P = 0.1, P = 0.72, and P = 0.06, respectively). A fold change of 1 was calculated in the control group in all experiments.

**Figure 4.**
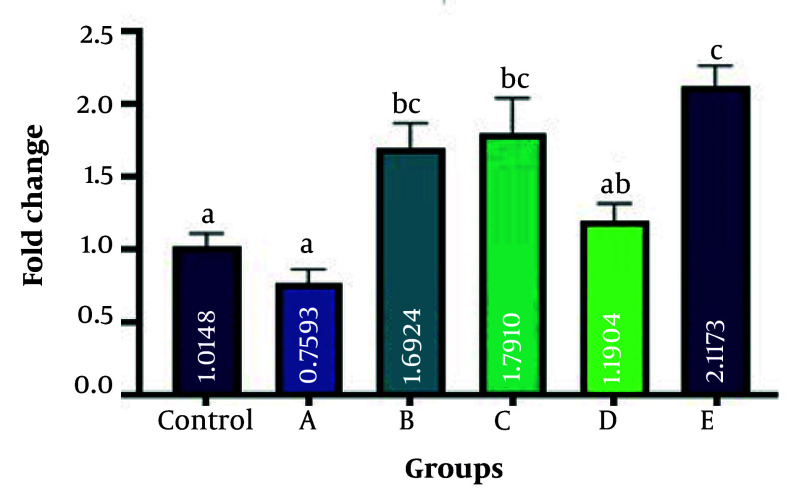
Evaluation of hormone-sensitive lipase (HSL) gene expression among the groups in comparison with the control group. There were significant increases in groups B, C, D, and E (P = 0.003, P = 0.003, P = 0.022, and P = 0.002, respectively). No difference in gene expression was observed in group A compared to the control (P = 0.42). Different letters indicate significant differences between treatments.

**Figure 5.**
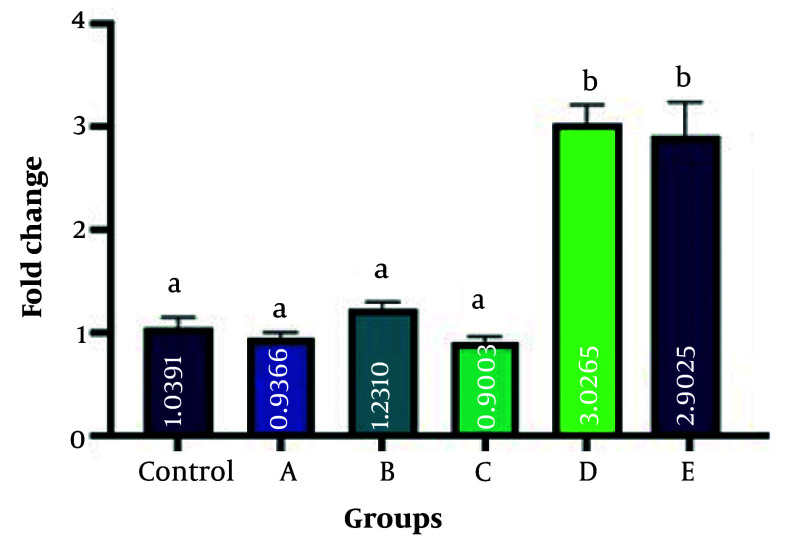
Evaluation of leptin gene expression among groups in comparison with the control group. The highest expression occurred in groups D and E compared to the control group (P = 0.005, and P = 0.006, respectively). No difference in gene expression was observed in groups A, B, and C, compared to the control (P = 0.052, P = 0.1, P = 0.72, and P = 0.06, respectively). Different letters indicate significant differences between treatments.

### 4.4. High Performance Liquid Chromatography Results

**Figure 6.**
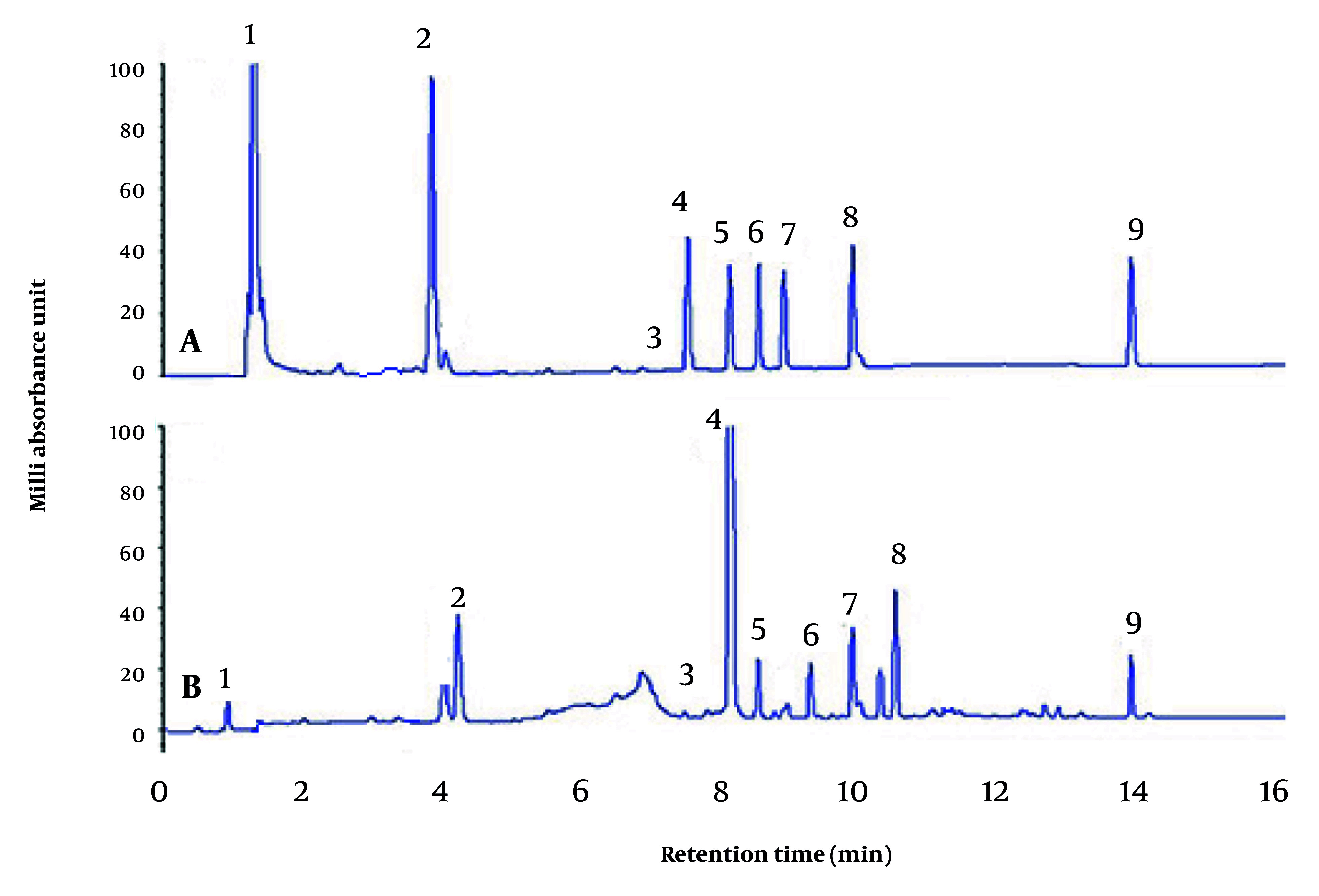
Separation of 9 ginsenosides in ginseng extract (GE) by high performance liquid chromatography (HPLC). A, Standard mixture containing ginsenosides. B, Ginseng extract. Peaks 1, Re; 2, Rg1; 3, Rf; 4, Rb1; 5, Rc; 6, Rb2; 7, Rd; 8, Rg3; and 9, CK ginsenosides.

A total of 9 ginsenosides consisting of Re, Rg1, Rf, Rb1, Rc, Rb2, Rd, Rg3, and CK were found in the GE with the retention times 3.79–17.43 min, among which Rb1 was present in the largest amount (100 mAU) (Figure 6). 

**Figure 7.**
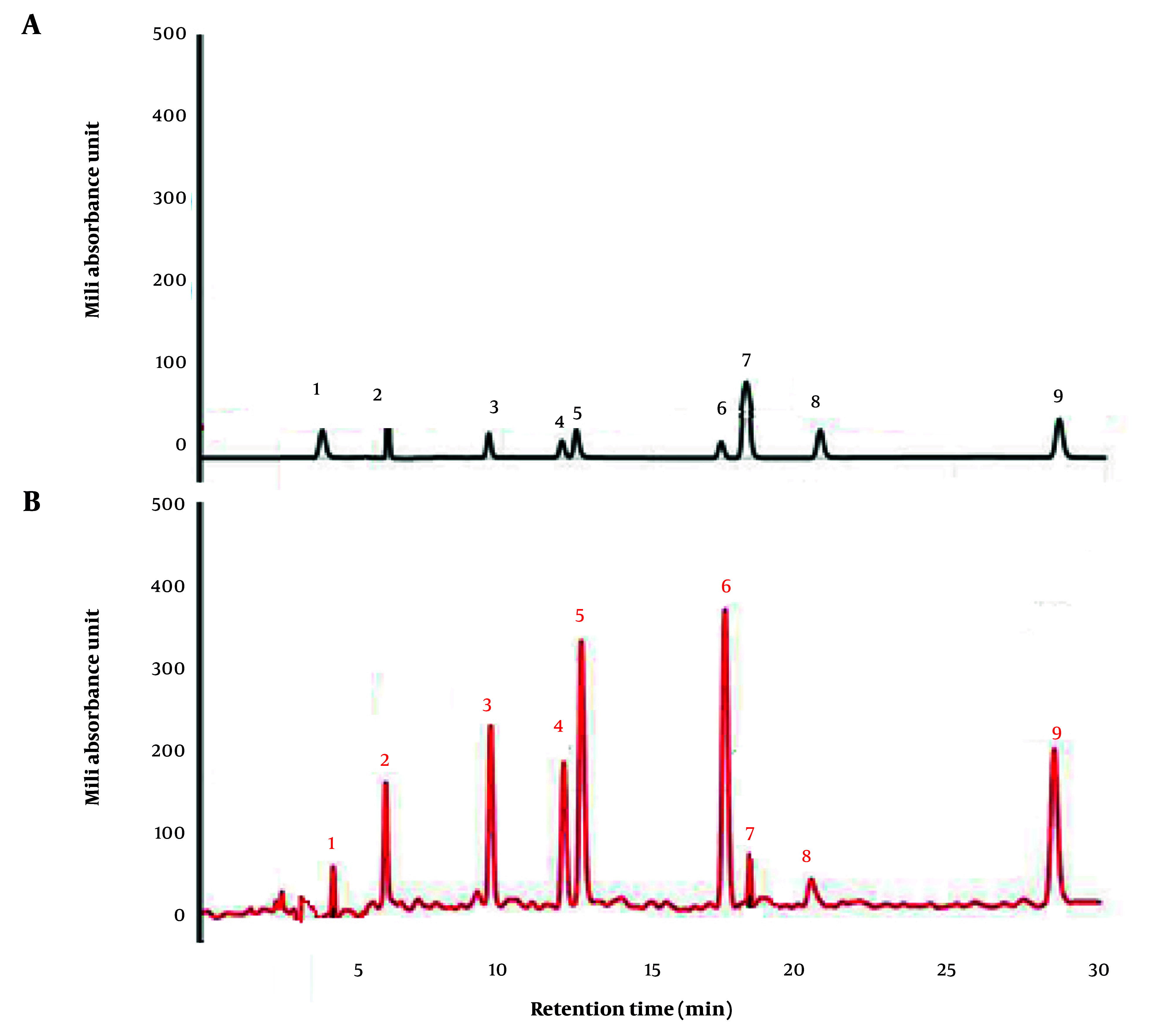
Separation of 8 catechins and one caffeine in green tea extract (GTE) by high performance liquid chromatography (HPLC). A, Standard mixture containing catechins and caffeine. B, Green tea extract. Peaks 1, GA; 2, GC; 3, EGC; 4, C; 5, Caf.; 6, EC; 7, EGCG; 8, GCG; and 9, ECG catechins.

In the GTE, a total of 8 catechins including catechin (C), epicatechin (EC), gallocatechin (GC), epigallocatechin (EGC), catechin gallate (CG), gallocatechin gallate (GCG), epicatechin gallate (ECG), and epigallocatechin gallate (EGCG) as well as one caffeine (Caf.) were found with the retention times 1.02–13.93. The major active alkaloids were EC (380 mAU) and Caf. (320 mAU) (Figure 7). 

## 5. Discussion

Different effects of ginseng and green tea have been studied in food science and nutritional research on humans and animals (27-30). In this study, we found that GE and GTE prevent obesity through weight loss and beneficial impact on serum parameters in obese rats. In addition, gene expression in groups A-E receiving GE and GTE was higher in converting WAT to BAT, particularly in group E with both extract concentrations.

The experimental rats subjected to GE and GTE exhibited significantly reduced levels of TG, TC, LDL, and VLDL, while concurrently displaying elevated levels of HDL in comparison to the control group. Elevated levels of TG, TC, and LDL alongside diminished HDL, frequently manifest in individuals afflicted by cardiovascular disease, a condition frequently linked to obesity (28), prompting an investigation into the potential impact of GTE on mitigating liver. Hence, the gut microbiota-targeted intervention holds the potential to exert an influential effect on HFD-induced obesity and avert consequent ailments stemming from such conditions. The impact of ingested food is subject to the prescribed dosage as indicated in previous studies (31, 32). The phenomenon of excessive consumption has been found to result in toxicity (30, 32), whereas an insufficient dosage has been associated with reduced efficacy (33, 34). According to the concentrations of 750 mg/day GTE that have been reported to significantly reduce body weight in humans (22, 24), in the present study, 77.2 mg/kg/day and 154.4 mg/kg/day of GTE were used for rats based on the formula from the US Food and Drug Administration. These dosages were also used considering the previous in vivo studies in which the dosages were determined based on the toxicity of active compounds such as catechins, present in the extracts (35, 36). On the other hand, a major challenge in research using medicinal plants is the required dosage to create synergy, if synergy can actually be achieved (36). Our results indicated that when treated mice were given 154.6 mg/kg/day GTE (group B), their body weight and BMI, serum biochemical parameters, and expression of fat burning genes had better levels than those of mice that received 77.2 mg/kg of the extract. Nevertheless, a lack of statistically significant disparity in obesity parameters was observable when comparing groups A and B. According to a recent study, GTE has been found to effectively activate peroxisome proliferator-activated receptor (PPAR), thereby triggering an anti-obesity mechanism and inducing the gene expression associated with the browning of WAT (9). According to the concentrations of 1000 mg/day GE that have been reported to significantly reduce body weight in humans (6, 24), in the current study, 102.8 mg/kg/day and 205.6 mg/kg GE were used for rats based on the formula from the US Food and Drug Administration. As our results indicated, treating mice with 205.6 was more effective on body weight and BMI, serum biochemical parameters, and expression of fat burning genes than that of mice that received 102.8 mg/kg/day GE. However, there was no statistically significant difference in obesity parameters between groups C and D, receiving 102.8 mg/kg/day and 205.6 mg/kg/day GE, respectively.

Previous studies suggest that brown fat is ideal for treating obesity due to its efficient fat burning and energy usage (37, 38). A preceding investigation documented the suppressive effects of increased expression of UCP-1 (uncoupling protein-1) in adipose tissue on obesity (39). Furthermore, as a consequence of the elevated expression of PRDM-16 and UCP-1 genes in BAT, it can be inferred that the augmentation of BAT activity confers resistance to weight gain (15, 40). This study presents the initial evidence of the efficacy of GE and GTE in activating BMP7, HSL, and leptin genes linked to the process of white fat browning. In another study, red GE reduced the weight and fat content of rats fed a high-calorie diet. It was also reported that this extract may be related to the regulation and reduction of leptin secretion and fat regulation (41). The intervention demonstrated a substantial mitigating impact on obesity in rats subjected to HFD. During the process of browning, the expression of the UCP-1 gene in WAT is amplified (16). The findings of our study suggest that GE and GTE may induce thermogenic browning of WAT, as evidenced by their ability to modulate key biomarkers associated with the browning process. Further investigations are required to affirm the impact of the examined extracts on the process of browning WAT across these specific anatomical regions. Previous investigations have documented a diminished level of messenger RNA expression of browning regulatory genes in WAT (42-44). Additional research is warranted to evaluate the potential impacts of GE and GTE on mice that are being subjected to disparate dietary regimens. The assessment of the anti-obesity effects of GE and GTE is commonly conducted through the examination of physical distinctions between the rats receiving the treatment and those in the control group. In the current investigation, GE and GTE elicited an upregulation in the expression of BMP7 and HSL genes. Consequently, this transcriptional activation resulted in an elevation in the browning process of WAT, thereby inducing the hydrolysis of lipid stores, ultimately leading to weight reduction. The findings imply that in rats subjected to HFD and treated with GE and GTE on a daily basis for a duration of eight weeks, WAT has a tendency to transition into BAT. In a recent scholarly investigation, Chen et al. (1) investigated the impact of green tea consumption on hyperlipidemic rats. The findings revealed significant weight loss, a notable upregulation in gene expression related to anti-obesity factors, and a discernible conversion of WAT into BAT. The results of their research were consistent with the findings presented in our study. In their investigation, rats subjected to HFD were administered GTE. Notably, GTE exhibited weight-reducing effects, ameliorated lipid-related serum parameters, and demonstrated improvement in obesity indicators among the experimental rat subjects.

It is believed that the health promotion activity of ginseng is due to the presence of the major class of bioactive compounds, ginsenosides (45, 46). In the present study, 9 ginsenosides consisting of Re, Rg1, Rf, Rb1, Rc, Rb2, Rd, Rg3, and CK were found in the GE; among which, Rb1 was present in the largest amount. Generally, Rb1 is abundant in roots. Other reports have shown that Rb1 is responsible for most of ginseng’s pharmacological properties, especially in the endocrine, cardiovascular, and immune systems (47). Also, it has been shown that bioactive flavonoids such as quercetin, catechin, hesperidin, and isorhamnetin may synergistically recover lipid metabolism by inducing the conversion of cholesterol to bile acids and cholesterol efflux, which leads to the inhibition of the de novo synthesis of cholesterol, and accelerating fatty acid oxidation (48).

In summary, our study has demonstrated the efficacy of the combination of GE and GTE, both independently and in combination, in augmenting biomarkers of BAT and possibly activating browning pathways within WAT. Additionally, these interventions have shown promise in mitigating the risk of HFD-induced obesity in hyperlipidemic rats, while follow-up experiments such as UCP-1 quantification, histological evidence of beige adipocytes, or metabolic assays are recommended. On the other hand, although the extract doses in the present study were determined in accordance with the doses that have been reported to significantly reduce body weight in humans, the need for controlled human clinical trials is suggested, especially given the variability in herbal products and differences in metabolism, dosage, and extract bioavailability in humans and animals.

### 5.1. Conclusions

The results of the current study showed that giving GE and GTE increased the expression of genes related to fatness reduction and resulted in significant weight loss over an eight-week period. The observations indicated that GE and GTE show potential as interventions for addressing obesity. While GE and GTE demonstrated significant effects on obesity in rats, further testing is needed, including extract standardization and utilization of larger sample sizes. Also, metabolic testing beyond gene expression toward mechanisms of action, protein-level validation, adipocyte morphology, thermogenesis assays, and identifying the extracts’ pharmacokinetics are required to optimize their therapeutic potential in humans.

## Data Availability

Raw data and protocols can be made available from the corresponding author upon reasonable request after publication.
